# Replication Study in Chinese Population and Meta-Analysis Supports Association of the 11q23 Locus with Colorectal Cancer

**DOI:** 10.1371/journal.pone.0045461

**Published:** 2012-09-18

**Authors:** Li Zou, Rong Zhong, Jiao Lou, Xuzai Lu, Qi Wang, Yang Yang, Jiahong Xia, Juntao Ke, Ti Zhang, Yu Sun, Li Liu, Yongping Cui, Haibing Xiao, Lei Chang, Ding Xia, Hua Xu

**Affiliations:** 1 Department of Urology, Tongji Hospital, Tongji Medical College, Huazhong University of Science and Technology, Wuhan, China; 2 Department of Epidemiology and Biostatistics and MOE Key Lab of Environment and Health, School of Public Health, Tongji Medical College, Huazhong University of Science and Technology, Wuhan, China; 3 Department of Cardiovascular Surgery, Union Hospital, Huazhong University of Science and Technology, Wuhan, China; 4 Department of Epidemiology and Biostatistics, School of Public Health, Guangdong Pharmaceutical University, Guangzhou, China; 5 Key Laboratory of Cellular Physiology, Ministry of Education, Shanxi Medical University, Taiyuan, Shanxi, China; Tongji Medical College, Huazhong University of Science and Technology, China

## Abstract

**Background:**

A common single nucleotide polymorphism (SNP), rs3802842, located at 11q23, was identified by genome-wide association studies (GWAS) to be significantly associated with the risk of colorectal cancer (CRC); however, the results of following replication studies were not always concordant. Thus, a case-control study and a meta-analysis were performed to clearly discern the effect of this variant in CRC.

**Method and Findings:**

We determined the genotypes of rs3802842 in 641 unrelated Chinese patients with CRC and 1037 cancer-free controls. Additionally, a meta-analysis comprising current and previously published studies was conducted. In our case-control study, significant associations between the polymorphism and CRC risk were observed in all genetic models, with an additive OR being 1.45 (95% CI = 1.26–1.67). The meta-analysis of 38534 cases and 39446 controls further confirmed the significant associations in all genetic models but with obvious between-study heterogeneity. Nevertheless, ethnicity, study type and whether subjects affected by Lynch syndrome could synthetically accounted for the heterogeneity. Besides, the cumulative and sensitivity analyses indicated the robust stability of the results.

**Conclusion:**

The results from our case-control study and meta-analysis provided convincing evidence that rs3802842 significantly contributed to CRC risk.

## Introduction

Colorectal cancer (CRC), as one of the most common malignancies, accounted for an estimated 1,230,000 new cases and 680,000 deaths worldwide in 2008 [1]. In United States, CRC was the second most commonly diagnosed cancer and the second leading cause of cancer death, with an incidence of 51.6 and a mortality of 19.7 per 100,000 population in 2008 [2]. In China, epidemiology studies indicate that the incidence rate of CRC has grown rapidly, especially in urban areas. The number of affected people increased as much as 4.2% every year from 1973 to 1993 in Shanghai, which was even higher than the global level (2%) [3]. Additionally, the data from 56 cancer registries in China showed that the incidence and mortality rates of CRC respectively ranked the third (31.4/100,000) and fifth (14.8/100,000) among the cancers that affected men and women in 2008 [4]. Among the risk factors for the disease, inherited susceptibility plays a role in the development of CRC, which is responsible for about 35% of variance in CRC risk [5]. However, high-penetrance germline mutations account for only 6% of CRC cases [6], suggesting that the remaining inheritance is likely to be a consequence of many common variants with low penetrance.

Genome-wide association studies (GWAS), efficiently applied to identify common genetic variants for complex diseases without prior knowledge of gene function, have so far uncovered multiple novel single nucleotide polymorphisms (SNPs) to CRC susceptibility [7]–[12]. Among these SNPs, rs3802842 (11q23.1), located in the intron region of *C11orf93*, was firstly identified in a GWA set of 3004 cases and 3094 controls and 8 replication sets of 14453 cases and 13259 controls [7]. Pittman AM et al. immediately validated the positive finding in the pooling data from 8 independent case-control series comprising a total of 10638 cases and 10457 controls [13]. Besides, rs3802842 is the first locus reported to exhibit a population difference between the Japanese and Caucasian populations [7]. Although there is much statistical evidence of this SNP for CRC, the results from replication studies are not always concordant [14]–[16], which is probably due to the so-called “winner’s curse” phenomenon that initial studies generally overestimate the effect sizes, replication studies are likely to be underpowered and so more likely to fail if the sample size calculations are based on the overestimated effect sizes [17]. Additionally, the modest effect of this variant may be also one of the important reasons for the failure of replication. Nevertheless, meta-analysis is a powerful technique to clarify the inconsistent findings in genetic association studies by increasing the sample size [18]. Notably, although the individual associated variants identified through GWAS confer only modest risks for CRC, the population attributable risk proportions have been considered to be significant because of substantial minor allele frequencies [19]. Thus, we conducted a replication study to examine the association between rs3802842 and CRC risk in a Chinese population using a case-control design, after that, a meta-analysis combining current and previously published studies about rs3802842 was further performed to provide a more precise estimate of this association. Then, the population attributable risk (PAR) was calculated in order to evaluate the effect of rs3802842 for CRC occurrence in general population.

## Materials and Methods

### Study Population

A total of 641 CRC cases were inpatients consecutively enrolled through the Tongji Hospital of Huazhong University of Science and Technology between 2009 and 2011, which is a top integrated hospital absorbing majority of cancer patients in Wuhan and nearby region. All cases were newly diagnosed and histopathologically confirmed without any treatment prior to blood samples collection. And 1037 cancer-free controls were selected randomly among the individuals who participated in health check-up programs at the same hospital in the same time period as the cases were enrolled. The health check up programs also primarily involved residents living in Wuhan and nearby region. The selection criteria for controls were no individual history of cancer and frequency matched to CRC cases on sex and age (±5 years). If a person was suspected to have CRC through the health check up programs and then histopathologically confirmed, he/she was selected as a case, whereas no case was ascertained through the health check up programs in this case-control study. All subjects were unrelated ethnic Han Chinese. At recruitment, epidemiologic data were collected by personal interview or a review of medical records, and 5-ml peripheral venous blood was drawn from each participant. Participants provided their written informed consent to participate in this study and this study was approved by ethnics committee of Tongji Hospital of Huazhong University of Science and Technology.

### Genotyping

Genomic DNA was extracted from 5-ml blood sample using the RelaxGene Blood System DP319-02 (Tiangen, Beijing, China) by reference to the manufacturer’s instructions. The rs3802842 was genotyped using the TaqMan SNP Genotyping Assay (Applied Biosystems, Fostercity, CA) on a 7900HT Fast Real-Time PCR System (Applied Biosystems, Fostercity, CA). To ensure quality control, 5% duplicated samples were randomly selected to assess the reproducibility, with a concordance rate of 100%.

### Statistical Analysis

Deviation of the genotype frequencies in controls from those expected under Hardy-Weinberg equilibrium was calculated using goodness-of-fit *χ^2^* test. Differences in demographic variables and distribution of genotypes between cases and controls were examined by *χ^2^* test or *t* test when appropriate. The association between rs3802842 and CRC risk was estimated as odds ratio (OR) with 95% confidence interval (95% CI), which was computed by unconditional multivariate logistic regression with adjustment for sex and age. ORs and 95% CIs as the metrics of effect size were recalculated for the allele C versus A, genotypes AC versus AA and CC versus AA. In order to avoid the assumption of genetic models, dominant, recessive and additive models were also analyzed. To adjust for multiple comparisons, the Bonferroni method was applied. Additionally, stratified analyses by tumor site and tumor differentiation were carried out to further evaluate the role of rs3802842 in CRC. All statistical analyses were performed in the SPSS 18.0 and all *P* values are two-tailed with a significant level at 0.05.

### Meta-analysis of rs3802842 in Association with CRC Risk

To ensure the rigour of this current meta-analysis, we designed and reported it according to the Preferred Reporting Items for Systematic Reviews and Meta-analyses (PRISMA) statement (http://www.prisma-statement.org) and the checklist is shown in Checklist S1.

We searched PubMed, EMBASE, and ISI Web of Science databases for studies published in any language up to April 2012 using the search terms *rs3802842*, *C11orf53*, or *11q23.1* combined with *colorectal cancer*, *colorectal neoplasia*, *colorectal adenoma*, *colon cancer*, or *rectal cancer*. To expand the coverage of our searches, we further performed searches in Chinese Biomedical (CBM) database [20] based on the above searching strategy. References of retrieved articles and reviews were also checked for additional studies. Searching was performed in duplicate by two independent reviewers (L. Zou and R. Zhong). The following criteria were applied for literature selection: (1) case-control study assessing the association between rs3802842 and CRC risk; (2) presentation of crude/adjusted OR with 95% CI or sufficient data to calculate crude/adjusted OR with 95% CI; (3) studies of humans. When there were multiple published reports from the same study population, the one with complete design or larger sample size was finally selected. If more than one ethnic population were included in one report, each population was considered separately. All data were extracted independently by two reviewers (L. Zou and R. Zhong) and any disagreement was adjudicated by a third author (Q. Wang). For each study, we summarized first author, year of publication, geographic location, ethnicity of study population, study type, genotyping method, numbers of cases and controls, male/female rate, mean age, family history of cancer, source of control group and frequencies of genotypes in cases and controls. The allele C versus A, genotypes AC versus AA and CC versus AA were all calculated and dominant, recessive and additive models were also assumed for rs3802842 respectively. The Bonferroni correction was also applied to counteract the problem of multiple comparisons. The between-study heterogeneity was assessed by *χ^2^-*based Cochran’s *Q* statistic (heterogeneity was considered significant at *P*<0.10). The *I^2^* statistic was then utilized to estimate heterogeneity quantitatively (*I^2^*<30%, no between-study heterogeneity or marginal between-study heterogeneity; *I^2^ = *30%–75%, mild heterogeneity; *I^2^*>75%, notable heterogeneity) [21]. A fixed-effects model, using Mantel-Haenszel method, was adopted to compute the pooled OR when no significant heterogeneity was detected [22]; otherwise, a random-effects model, using DerSimonian and Laird method, was applied [23]. Overall meta-analysis for rs3802842 was initially performed. Then we conducted stratification analyses if data permitted (the number of studies obtained in each subgroup is not less than 3), according to ethnicity (European, Asian and African), study type (GWAS and replication studies), tumor site (colon and rectum cancers) and whether subjects affected by Lynch syndrome. Additionally, sensitivity analysis was carried out, in which the pooled ORs were calculated after omission of each study in turn [24]. Cumulative meta-analysis was also applied through assortment of studies with publication time [25]. An estimation of potential publication bias was executed by Egger’s test [26]. If there was an significant association between the polymorphism and CRC risk detected in the overall meta-analysis for any genetic model, bioinformatics analyses were further carried out to predict the function of rs3802842 using three integrated bioinformatics tools “SNP Info” (http://manticore.niehs.nih.gov/snpfunc.htm), “FastSNP” (http://fastsnp.ibms.sinica.edu.tw/pages/input_CandidateGeneSearch.jsp) and “F-SNP” (http://compbio.cs.queensu.ca/F-SNP/). To examine the contribution of this polymorphism to the occurrence of CRC in general population, the PAR was computed using the following formula: Pr (RR-1)/[1+Pr (RR-1)], where *Pr*, the proportion of control subjects exposed to the allele of interest, can be calculated by minor allele frequency (MAF) reported in dbSNP database and the relative risk (RR) is estimated using the pooled OR produced by the meta-analysis [27]. All statistical analyses were performed with Stata 11.0 software and *P* values less than 0.05 are considered statistically significant for all tests except for *Q* test for heterogeneity.

## Results

### Results of Case-control Study

#### Population characteristics

The characteristics of cases and controls are listed in Table 1. No significant differences were found between cases and controls for sex and age. Males were 59.9% among cases compared with 59.1% among controls (*P* = 0.748). The mean age (±standard deviation) was 56.31 (±12.59) years for cases and 57.24 (±10.86) years for controls (*P* = 0.119). Of the cases, 250 had colon cancer and 391 had rectal cancer. For tumor differentiation, 80, 445 and 116 cases were classified as poorly, moderately and well differentiated respectively.

**Table 1:** The characteristics of the study population.

Variables	Case (N = 641) No. (%)	Control (N = 1037) No. (%)	*P*
Sex			0.748^b^
Male	384 (59.9)	613 (59.1)	
Female	257 (40.1)	424 (40.9)	
Age	56.31±12.59^a^	57.24±10.86^a^	0.119^c^
Tumor site			
Colon	250 (39.0)		
Rectum	391 (61.0)		
Tumor differentiation			
Poorly	80 (12.5)		
Moderately	445 (69.4)		
Well	116 (18.1)		

a Age was represented as mean±standard deviation.

b *P* value was calculated by the *x* ^2^ test.

c *P* value was calculated by the *t* test.

#### Association analysis

The genotype data of rs3802842 for cases and controls are shown in Table 2. The genotype distribution in controls complied with Hardy-Weinberg equilibrium (*P* = 0.323). The genotype frequencies for this polymorphism in the case group differed from those in the control group (*P* = 0.000). In the multivariate logistic regression model adjusted for age and sex, individuals who carried the C allele, AC or CC genotype had significantly elevated risks of CRC compared with those carried the A allele or AA genotype (C versus A: OR = 1.44, 95% CI = 1.25–1.66; AC versus AA: OR = 1.76, 95% CI = 1.40–2.21; CC versus AA: OR = 1.99, 95% CI = 1.48–2.67). Likewise, significant associations between this polymorphism and CRC risk were found in dominant, recessive and additive models (dominant model: OR = 1.82, 95% CI = 1.46–2.26; recessive model: OR = 1.41, 95% CI = 1.09–1.82; additive model: OR = 1.45, 95% CI = 1.26–1.67). The association between rs3802842 and risk of CRC for all comparisons and genetic models remained significant after correction for multiple testing.

**Table 2:** Association between rs3802842 and colorectal cancer risk in a Chinese population^a^.

	Case	Control	OR (95% CI)					
	AA/AC/CC	AA/AC/CC	C VS. A	AC VS. AA	CC VS. AA	Dominant model	Recessive model	Additive model
Total	163/345/133	397/477/163	1.44 (1.25–1.66)	1.76 (1.40–2.21)	1.99 (1.48–2.67)	1.82 (1.46–2.26)	1.41 (1.09–1.82)	1.45 (1.26–1.67)
Tumor site								
Colon	52/148/50	397/477/163	1.56 (1.28–1.89)	2.37 (1.68–3.34)	2.34 (1.52–3.59)	2.36 (1.70–3.28)	1.34 (0.94–1.91)^b^	1.57 (1.28–1.91)
Rectum	111/197/83	397/477/163	1.38 (1.17–1.63)	1.47 (1.12–1.92)	1.85 (1.32–2.59)	1.57 (1.22–2.02)	1.47 (1.09–1.98)^b^	1.37 (1.16–1.62)
Tumor differentiation								
Poorly+Moderately	137/280/108	397/477/163	1.42 (1.22–1.65)	1.70 (1.33–2.17)	1.92 (1.41–2.63)	1.76 (1.39–2.21)	1.39 (1.06–1.82)^b^	1.42 (1.22–1.65)
Well	26/65/25	397/477/163	1.56 (1.18–2.05)	2.03 (1.26–3.26)	2.39 (1.33–4.27)	2.12 (1.34–3.34)	1.53 (0.95–2.46)^b^	1.56 (1.18–2.05)

Abbreviations: OR, Odds ratio; 95% CI, 95% confidence interval.

a ORs and their corresponding 95% CIs were calculated by multivariate logistic regression model after adjusting for age and sex.

b The association between rs3802842 and colorectal cancer risk turned to be null significant after Bonferroni correction for multiple testing was applied.

We then stratified data according to the pathological factors (Table 2). The polymorphism was associated with increased risk of colon cancer in all genetic models except the recessive model. Significant associations between the polymorphism and rectum cancer for all genetic models were also observed. However, the adjustment for multiple testing resulted in null-significant association in recessive model for colon or rectum cancer. Regarding tumor differentiation, the variant in all genetic models presented significantly elevated risk of CRC with poorly or moderated differentiated. For well differentiated cancer, apart from the recessive model, significant associations between the polymorphism and CRC risk were detected. After correction for multiple testing, the association was observed to be null significant in recessive model for poorly, moderated or well differentiated cancer.

### Results of Meta-analysis

#### Study characteristics

Figure S1 shows the literature search and study selection procedures. After comprehensive searching, 31 potentially relevant publications were identified and screened for retrieval, of which, 15 publications met the inclusion criteria. However, the publications respectively reported by Hutter CM et al. [28], Mates IN et al. [29], Niittymäki I et al. [30], and Lubbe SJ et al. [31] were excluded since the cases largely overlapped with the samples of previous studies. Therefore, 11 publications plus current study comprising 32 case-control studies of 38534 cases and 39446 controls contributed data for this meta-analysis [7], [13]–[16], [32]–[37]. Among these, the publications respectively reported by Kupfer SS et al. [15] and He J et al. [16] just provided adjusted additive ORs, thus were merely included in the pooled analysis for additive model. Besides, there was only an allelic OR obtained in the publication by Middeldorp A et al. [32], hence the study merely participated in the pooled analysis for allelic model. In addition, 3 studies included some Lynch syndrome patients [32], [33], [35]. Table 3 shows the characteristics of the included studies.

**Table 3:** The characteristics of included studies.

First author	Year	Country	Ethnicity	Study type	Genotyping method	Case/control	Cases' characteristics			Controls' characteristics		
							Male/female ratio	Mean age (SD)	Family history of cancer	Male/female ratio	Mean age (SD)	Control source
Tenesa A (Scotland 1)	2008	Scotland	European	GWAS	Illumina	3004/3094	–	–	–	–	–	Population based
Tenesa A (Scotland 2)	2008	Scotland	European	Replication	TaqMan	937/941	–	–	–	–	–	Population based
Tenesa A (Canada)	2008	Canada	European	Replication	TaqMan, Sequenom	1175/1184	–	–	–	–	–	Population based
Tenesa A (DACHS)	2008	German	European	Replication	TaqMan	1373/1480	–	–	–	–	–	Population based
Tenesa A (England)	2008	England	European	Replication	TaqMan	2253/2262	–	–	–	–	–	Population based
Tenesa A (Israel)	2008	Israel	European	Replication	TaqMan	1789/1771	–	–	–	–	–	Population based
Tenesa A (Japan)	2008	Japan	Asian	Replication	Invader assay	4400/3179	–	–	–	–	–	–
Tenesa A (Kiel)	2008	German	European	Replication	TaqMan	2169/2145	–	–	–	–	–	Population based
Tenesa A (Spain)	2008	Spain	European	Replication	TaqMan	357/297	–	–	–	–	–	Hospital based
Pittman AM (CORGI)	2008	England	European	GWAS	Illumina	619/932	0.82	–	Yes	0.87	–	Population based
Pittman AM (DFCCS)	2008	Netherlands	European	Replication	KASPar	783/664	0.90	53.4 (13.4)	Yes	0.61	51.1 (11.3)	Population based
Pittman AM (EPICOLON)	2008	Spain	European	Replication	Sequenom iPLEX	515/515	1.45	70.6 (11.3)	No	1.29	69.8 (11.7)	Hospital based
Pittman AM (FCCPS)	2008	Finland	European	Replication	KASPar	1001/1034	1.03	67.4 (11.8)	–	–	–	Population based
Pittman AM (MCCS)	2008	Australia	European	Replication	HRM	515/709	1.10	66.2 (7.7)	–	0.99	57.9 (7.0)	Population based
Pittman AM (NSCCG1)	2008	England	European	GWAS	Illumina	2863/2838	0.72	59.3 (8.7)	–	0.67	59.8 (10.8)	Population based
Pittman AM (NSCCG2)	2008	England	European	Replication	KASPar	3036/2944	1.16	59.4 (8.2)	–	0.67	55.2 (12.3)	Population based
Pittman AM (VCQ)	2008	England	European	Replication	KASPar,	1251/799	0.89	–	–	0.73	–	Population based
Middeldorp A^ac^	2009	Netherlands	European	Replication	KASPar	995/1340	0.92	–	Yes	0.90	–	Population based
Wijnen JT^a^	2009	Netherlands	European	Replication	KASPar	131/544	1.43	–	–	0.70	–	Population based
Kupfer SS (Combined African-American)^b^	2010	America	African	Replication	MassARRAY	795/985	0.88	64.5 (11.7)	–	0.72	62.3 (13.2)	Population based
Kupfer SS (European-American)^b^	2010	America	European	Replication	MassARRAY	399/367	1.35	64.6 (13.1)	–	1.01	61.1 (12.7)	Population based
Von Holst S	2010	Sweden	European	Replication	DeCode test	1786/1749	1.13	68.6	22%^d^	–	66.3	Population based
Xiong F	2010	China	Asian	Replication	PCR-RFLP	2124/2124	1.48	56.9 (11.8)	–	1.4	56.4 (11.3)	Population based
Talseth-Palmer BA^a^	2010	Australia,Poland	European	Replication	Real-Time PCR	262/314	–	–	–	–	–	Hospital based
He J (European American)^b^	2011	America	European	Replication	TaqMan	1171/1534	–	–	–	–	–	–
He J (African American)^b^	2011	America	African	Replication	TaqMan	382/510	–	–	–	–	–	–
He J (Native Hawaiian)^b^	2011	America	European	Replication	TaqMan	323/472	–	–	–	–	–	–
He J (Japanese American)^b^	2011	America	Asian	Replication	TaqMan	1042/1426	–	–	–	–	–	–
He J (Latinos)^b^	2011	America	European	Replication	TaqMan	393/524	–	–	–	–	–	–
Ho JW	2011	HK, China	Asian	Replication	Sequenom	892/890	1.64	66.75 (12.25)	–	–	–	Hospital based
Mates IN	2012	Romania	European	Replication	DeCode test	153/182	0.99	67.9 (11.2)	15.7% d	3.92	60.8 (14.7)	Hospital based
Current study	2012	China	Asian	Replication	TaqMan	641/1037	1.49	56.31 (12.59)	–	1.45	57.24 (10.86)	Population based

Abbreviations: GWAS, genome-wide association studies; SD, standard deviation; HRM, high resolution melt curve analysis.

a The study included some Lynch syndrome patients.

b The study only provided an adjusted additive OR.

c The study only provided an allelic OR.

d The percentage of patients having a family history of cancer.

#### Overall meta-analysis of rs3802842 in associated with CRC

As shown in Table 4, significant evidence of heterogeneity was detected in all genetic models (all *P* for heterogeneity <0.10), therefore ORs for all genetic models were pooled under random-effects model. Compared to the A allele, the C allele conferred a pooled OR of 1.15 (95% CI = 1.11–1.19) in the allelic model. Genotypic ORs of the AC versus AA and CC versus AA were 1.18 (95% CI = 1.12–1.24) and 1.31 (95% CI = 1.20–1.42), respectively. Similarly, significant associations between this polymorphism and CRC risk were found in dominant, recessive and additive models (dominant model: OR = 1.20, 95% CI = 1.15–1.26; recessive model: OR = 1.20, 95% CI = 1.12–1.29; additive model: OR = 1.15, 95% CI = 1.11–1.19). The additive OR and corresponding 95% CI did not change after both crude and adjusted ORs were combined (OR = 1.15, 95% CI = 1.11–1.19). The positive results remained significant after adjusted by multiple testing.

**Table 4:** Meta-analysis of the rs3802842 in association with colorectal cancer risk^a^.

	Genetic model	OR (95% CI)	*P*	*I* ^2^ (%)	*P*_heterogeneity_	*P* for Egger’s test
Overall						
Overall (n = 25)	C VS. A	1.15 (1.11–1.19)	0.000	49.6%	0.003	0.533
Overall (n = 24)	AC VS. AA	1.18 (1.12–1.24)	0.000	46.3%	0.007	0.216
	CC VS. AA	1.31 (1.20–1.42)	0.000	51.3%	0.002	0.968
	Dominant model	1.20 (1.15–1.26)	0.000	54.0%	0.001	0.283
	Recessive model	1.20 (1.12–1.29)	0.000	37.2%	0.036	0.818
	Additive model	1.15 (1.11–1.19)	0.000	47.2%	0.006	0.568
Overall (n = 31)	Additive model^b^	1.15 (1.11–1.19)	0.000	43.2%	0.006	0.499
Ethnicity						
European (n = 21)	C VS. A	1.14 (1.11–1.18)	0.000	8.4%	0.349	0.934
European (n = 20)	AC VS. AA	1.15 (1.11–1.20)	0.000	0.0%	0.457	0.665
	CC VS. AA	1.30 (1.20–1.40)	0.000	20.9%	0.195	0.482
	Dominant model	1.18 (1.14–1.22)	0.000	0.0%	0.467	0.841
	Recessive model	1.21 (1.12–1.31)	0.000	24.5%	0.155	0.432
	Additive model	1.13 (1.09–1.17)	0.000	29.3%	0.108	0.890
Asian (n = 4)	C VS. A	1.20 (1.02–1.38)	0.000	88.1%	0.000	0.443
	AC VS. AA	1.33 (1.07–1.66)^c^	0.011	86.6%	0.000	0.210
	CC VS. AA	1.39 (1.03–1.87)^c^	0.033	87.1%	0.000	0.518
	Dominant model	1.35 (1.06–1.71)^c^	0.014	90%	0.000	0.272
	Recessive model	1.16 (0.97–1.39)^c^	0.100	71%	0.016	0.801
	Additive model	1.22 (1.09–1.36)	0.000	73.2%	0.011	0.428
Study type						
GWAS (n = 3)	C VS. A	1.20 (1.14–1.27)	0.000	0.0%	0.948	0.204
	AC VS. AA	1.17 (1.09–1.26)	0.000	0.0%	0.571	0.535
	CC VS. AA	1.49 (1.33–1.68)	0.000	0.0%	0.463	0.428
	Dominant model	1.22 (1.14–1.31)	0.000	0.0%	0.882	0.607
	Recessive model	1.40 (1.23–1.59)	0.000	21.3%	0.281	0.453
	Additive model	1.20 (1.14–1.26)	0.000	0.0%	0.903	0.275
Replication (n = 22)	C VS. A	1.14 (1.10–1.19)	0.000	50.2%	0.004	0.376
Replication (n = 21)	AC VS. AA	1.18 (1.13–1.24)	0.000	52.1%	0.003	0.163
	CC VS. AA	1.27 (1.16–1.39)	0.000	48.5%	0.007	0.798
	Dominant model	1.20 (1.13–1.28)	0.000	59.0%	0.000	0.190
	Recessive model	1.15 (1.08–1.24)	0.000	21.0%	0.190	0.936
	Additive model	1.14 (1.09–1.19)	0.000	48%	0.008	0.380
Subjects with/without Lynch syndrome						
With Lynch syndrome (n = 3)	C VS. A	1.22 (1.08–1.35)	0.000	0.0%	0.589	0.355
Without Lynch syndrome (n = 22)	C VS. A	1.16 (1.11–1.20)	0.000	56.2%	0.001	0.525
	AC VS. AA	1.18 (1.12–1.24)	0.000	50.3%	0.004	0.167
	CC VS. AA	1.31 (1.20–1.43)	0.000	55.5%	0.001	0.957
	Dominant model	1.21 (1.15–1.27)	0.000	57.6%	0.000	0.224
	Recessive model	1.20 (1.12–1.29)	0.000	42.5%	0.019	0.860
	Additive model	1.15 (1.11–1.19)	0.000	51.5%	0.003	0.492

a Crude ORs were combined in a fixed- or random- effects model.

b Both crude and adjusted ORs were combined.

c The association between rs3802842 and colorectal cancer risk turned to be null significant after Bonferroni correction for multiple testing was applied.

#### Stratified analysis

The stratified analysis was firstly conducted by ethnicity (Table 4). Because of less than 3 studies regarding African, the specific data for rs3802842 were only stratified into two subgroups: European and Asian. After stratifying by ethnicity, significant between-study heterogeneity was effectively reduced in European, whereas the heterogeneity in Asian was still detected. In European population, the polymorphism presented significantly increased risks of CRC in all genetic models. In Asian population, all genetic models except the recessive model exhibited significant associations with CRC risk. However, no significant association was found for the genotype AC versus AA, CC versus AA, dominant model and recessive model when the Bonferroni correction was performed. The data were further stratified by study type into GWAS and replication studies (Table 4). Heterogeneity for all genetic models was not detected in the subgroup of GWAS, however, there was significant heterogeneity in the subgroup of replication studies. Statistically significant findings for all genetic were seen either in the GWAS or in the replication studies. The data were additionally stratified into subgroup of subjects affected or not affected by Lynch syndrome (Table 4). All pooled ORs except the allelic OR could not be appraised in the subjects affected by Lynch syndrome due to limited number of studies. Significant association between the variant and CRC risk was observed without heterogeneity in the allelic model. In terms of subjects not affected by Lynch syndrome, there was evidence of heterogeneity in all genetic models and significant associations of CRC risk with the polymorphism were found. The stratified analysis by tumor site could not be performed owing to lack of data.

#### Sensitivity analysis

Since significant between-heterogeneity for the overall meta-analyses was observed in all genetic models, we performed sensitivity analyses in an attempt to assess the effects of each individual study on the pooled OR under random-effects model. As shown in Table S1, S2, S3, S4, S5, S6, all of the results were not materially altered and did not draw different conclusion, suggesting that our results were robust.

#### Cumulative meta-analysis

Cumulative meta-analyses were also conducted in all genetic models via assortment of studies in chronologic order. As shown in Figure S2, the plots made it clear that although the studies reduced the 95% CI for the summary estimates, they did not change the inclinations toward significant associations.

#### Publication bias

The results of Egger’s test indicated that no evidence of publication bias was observed in all genetic models (all *P*>0.05).

#### The bioinformatics analysis and PAR of rs3802842

All of the three bioinformatics tools conformably predicted that the SNP might change the transcription factor binding sites of *C11orf93*. In order to evaluate the percent of the incidence of a disease in the population that is due to exposure, the PAR was calculated. The MAF of rs3802842 (C allele) was 31.3% and the pooled OR of overall meta-analysis in additive model was 1.15, so the PAR for C allele was estimated to be 4.5%.

## Discussion

The present study demonstrated an association between rs3802842 and increased risk of CRC in a Chinese population. Then, the following meta-analysis based on 32 case-control studies of 38534 cases and 39446 controls also suggested that the SNP was significantly associated with CRC risk, with an additive OR being 1.15. Accordingly, PAR which takes into account both magnitude of the risk and risk allele frequency in the general population was 4.5%. Cumulative analysis in chronologic order further confirmed the positive findings, exhibiting that the effect of this variant progressively significant with more precise estimation. In addition, sensitivity analyses indicated the stability of the result. To the best of our knowledge, this is the first meta-analysis seeking to clarify the association between rs3802842 and risk of CRC and the results strongly emphasize the role of this polymorphism in CRC susceptibility.

rs3802842 is located at 11q23 and there are four ORFs (*LOC120376*, *FLJ45803*, *C11orf53* and *POU2AF1*) and a SNP (rs12296076) recognized as polymorphic binding site target for miRNAs in high linkage disequilibrium around rs3802842 within a range of 100 kb [7]. Moreover, rs3802842 is near the genes encoding POU transcription factors [7]. Thus it can be seen that the region where rs3802842 is located doubtlessly adds difficulty and complexity to discern the role of this polymorphism in CRC. Little is known about the function of rs3802842, but our bioinformatics analyses indicated that it was likely to alter the transcription factor binding sites of *C11orf93* and further affected the expression of the gene, whereas the function of *C11orf93* also remains elusive. Besides, albeit the important role of the region 11q23 in the pathogenesis of CRC has been confirmed [38], Pittman AM et al. inferred that the potential genomic sequence change caused by rs3802842 might affect the expression of genes mapping outside 11q23.1 through *cis*- or *trans*- regulatory [13]. Alternatively, most of the variants identified by GWAS cannot be *per se* causal but imply the probability of being in linkage with the “real” causal variants [19], so it is possible that the polymorphism is in linkage disequilibrium with “real” causal loci which are hitherto uncharacterized. However, the achievements of fine-mapping the causal variants responsible for GWAS signals which have been largely predicated on common disease common variant theory remain limited, one reason for this may be that a number of rare variants which are not identified by GWAS account for much of the remaining heritability of diseases, reflecting the important role of rare variants in the risk of disease occurrence [39]. Moreover, understanding the biological function of risk loci, with a focus on non-coding variants, is the greatest challenge in the post-GWAS era [40].

After the first GWAS with respect to rs3802842, multiple replication studies were carried out in succession with inconsistent results. Our case-control study found a significant association between the polymorphism and CRC risk in all genetic models, which was consistent with the GWAS and some replication studies [7], [13], [34]. Stratified analyses by tumor site or differentiation showed similar results, but not statistically significant in recessive model, which may be due to the sample size or inheritance pattern. Despite positive findings corroborated in our meta-analysis, obvious between-study heterogeneity cannot be ignored; hence stratified analyses were conducted to dig out the source of heterogeneity. When stratified by ethnicity, heterogeneity was greatly reduced in European but not in Asian, implying that there might be varied manners of action and different allele frequencies of rs3802842 between the two populations. Besides, it was noted that Tenesa A et al. observed significantly different allelic effects of rs3802842 between the Japanese and Europeans and further discovered that the population difference was site-specific, that is, the Japanese population showed the variant only associated with the increased risk of rectal cancer, but not associated with the risk of colon cancer that was detected in European population [7]. However, whether colon or rectal cancer risk was related to the polymorphism in our case-control study, suggesting that notwithstanding the same ethnicity the Chinese and Japanese both belong to, the two populations may differ in the causes of CRC to some extent. Regarding study type, heterogeneity disappeared in GWS studies but still remained in replication studies, which was resulted from the diverse genotyping methods. After stratifying by whether subjects affected by Lynch syndrome, heterogeneity was almost removed in the subgroup of subjects affected by Lynch syndrome but did not change in the other subgroup, naturally reflecting the different attributes of research subjects. Taken together, ethnicity, study type and whether subjects affected by Lynch syndrome were likely to be the sources of heterogeneity in this meta-analysis.

Despite the clear strengthen of this study that conducted a case-control study and a meta-analysis at the same time to substantiate the association between rs3802842 and CRC risk, some limitations should be acknowledged. First, the sample size of our case-control study was relatively small. Second, we merely performed bioinformatics analyses rather than functional experiments, so whether this variant is causal remained uncertain. Third, CRC is a complex disease related to environmental and genetic factors. However, in this study, only genetic factor was taken into consideration which restricted to explore the gene-environment interaction.

In conclusion, our case-control study and the following meta-analysis provided convincing evidence for the genetic involvement of rs3802842 polymorphism in CRC susceptibility. However, it is needed to carry out fine-mapping of 11q23 region and functional experiments to identify causal loci.

## Supporting Information

Figure S1 **Flow diagram of the study selection procedure.**
(TIF)

Figure S2 **Forest plots of cumulative meta-analysis of rs3802842 in association with colorectal cancer by published year under different genetic models.** (A) the C versus A; (B) the AC versus AA; (C) the CC versus AA; (D) the dominant model; (E) the recessive model; (F) the additive model.
(TIF)

Table S1 **Sensitivity analysis of allelic model.**
(DOCX)

Table S2 **Sensitivity analysis of the genotypic model of AC versus AA.**
(DOCX)

Table S3 **Sensitivity analysis of the genotypic model of CC versus AA.**
(DOCX)

Table S4 **Sensitivity analysis of dominant model.**
(DOCX)

Table S5 **Sensitivity analysis of recessive model.**
(DOCX)

Table S6 **Sensitivity analysis of additive model.**
(DOCX)

Checklist S1 **The PRISMA 2009 Checklist.**
(DOC)
