# Supplementary figures and images for: Replication Study in Chinese Population and Meta-Analysis Supports Association of the 11q23 Locus with Colorectal Cancer
Source: PLoS One. 2012 Sep 18;7(9):e45461. doi: 10.1371/journal.pone.0045461 (PMC3445543; doi:10.1371/journal.pone.0045461)

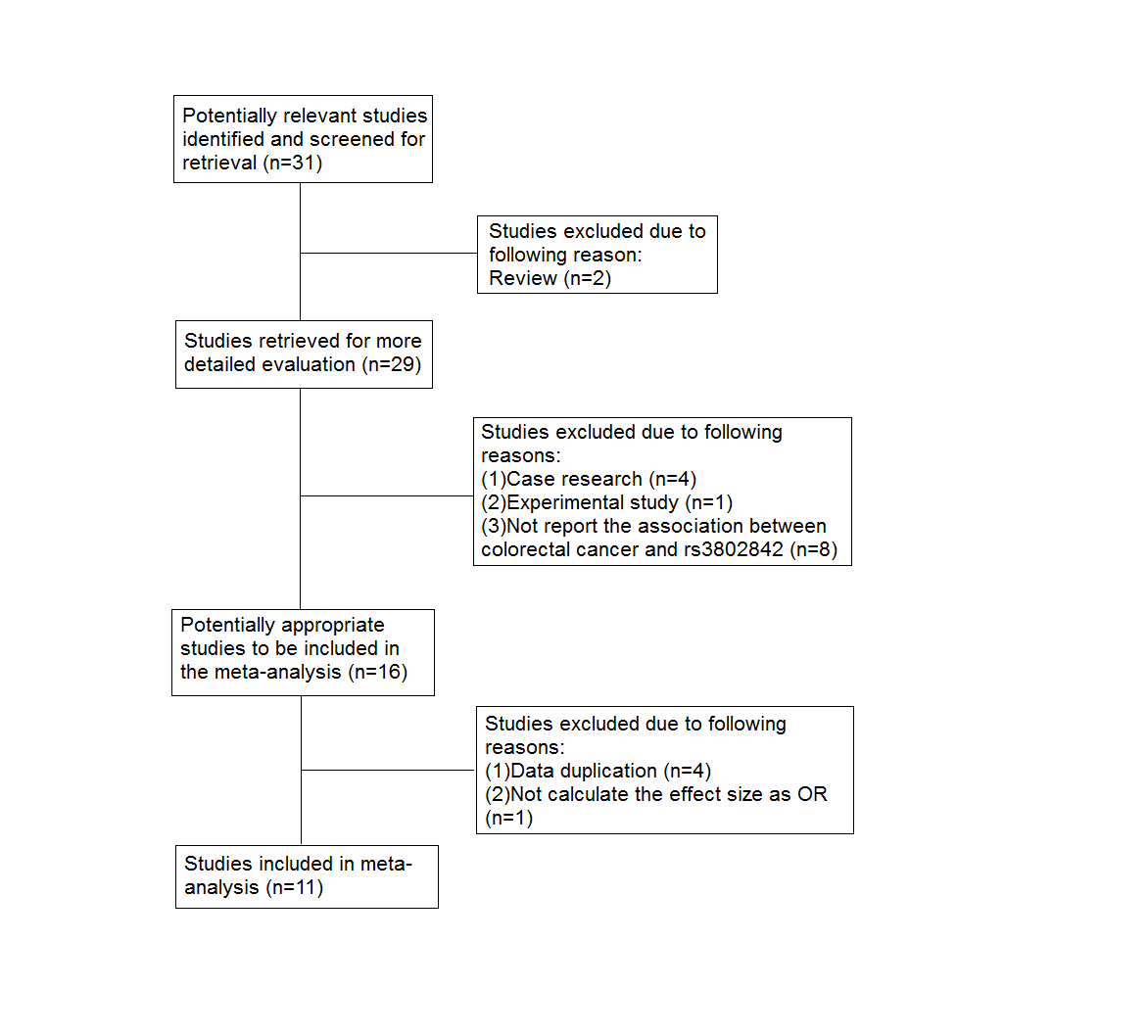

Supplement: Figure S1 — Flow diagram of the study selection procedure. (TIF) [file pone.0045461.s001.tif]

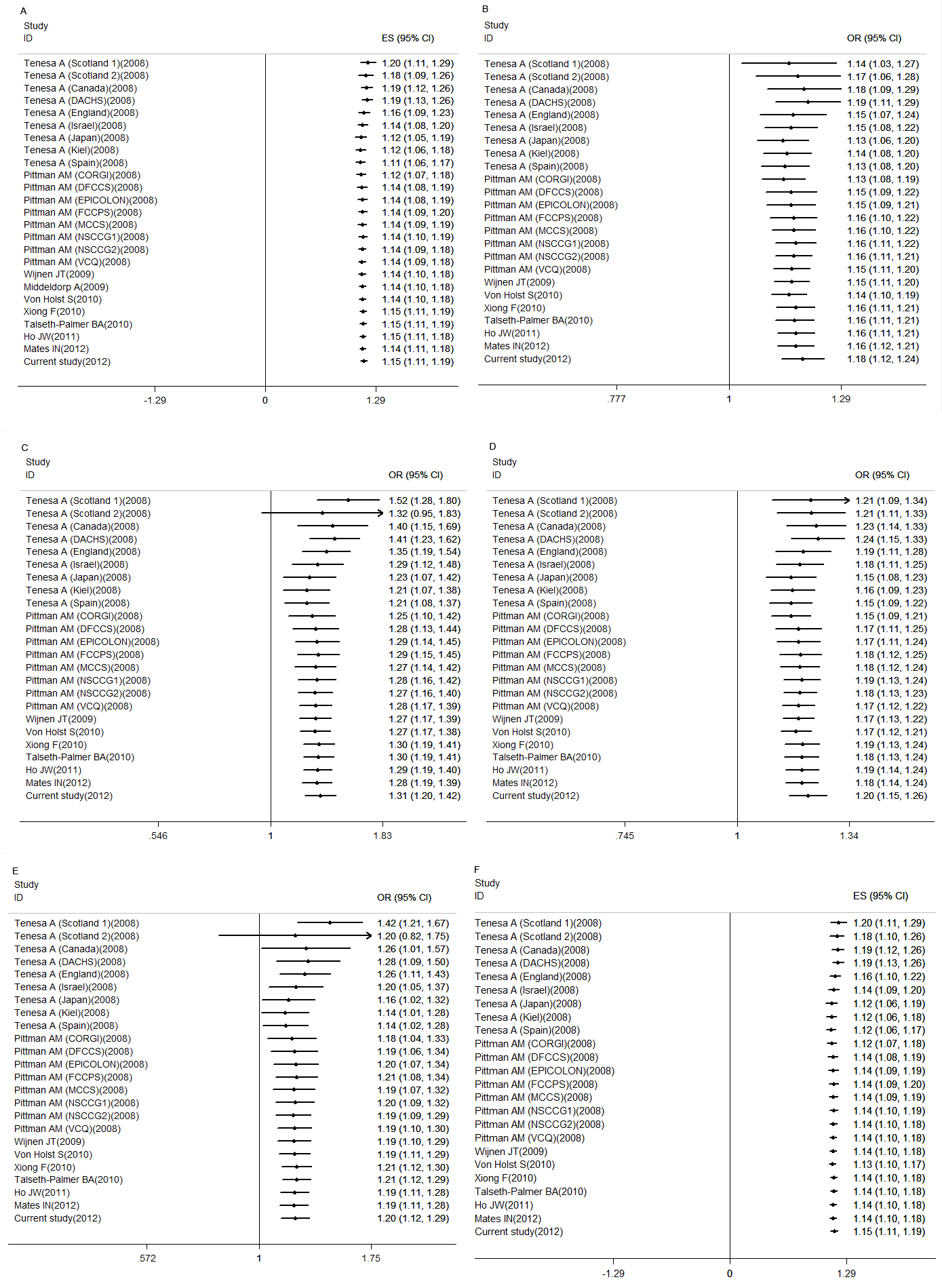

Supplement: Figure S2 — Forest plots of cumulative meta-analysis of rs3802842 in association with colorectal cancer by published year under different genetic models. (A) the C versus A; (B) the AC versus AA; (C) the CC versus AA; (D) the dominant model; (E) the recessive model; (F) the additive model. (TIF) [file pone.0045461.s002.tif]
