# Supplementary material for: Replication Study in Chinese Population and Meta-Analysis Supports Association of the 11q23 Locus with Colorectal Cancer
Source: PLoS One. 2012 Sep 18;7(9):e45461. doi: 10.1371/journal.pone.0045461 (PMC3445543; doi:10.1371/journal.pone.0045461)
Supplement: Table S1 — Sensitivity analysis of allelic model. (DOCX) [file pone.0045461.s003.docx]

**Table S1.** Sensitivity analysis of allelic model.

| Omitted study | OR (95%CI) | *P*^a^ | *P*^b^_heterogeneity_ | *I*^2^(%) |
| --- | --- | --- | --- | --- |
| Tenesa A (Scotland 1) | 1.15 (1.11-1.19) | 0.000 | 0.004 | 49.3 |
| Tenesa A (Scotland 2) | 1.16 (1.11-1.20) | 0.000 | 0.002 | 51.5 |
| Tenesa A (Canada) | 1.15 (1.11-1.19) | 0.000 | 0.003 | 50.2 |
| Tenesa A (DACHS) | 1.15 (1.11-1.19) | 0.000 | 0.002 | 50.8 |
| Tenesa A (England) | 1.16 (1.12-1.20) | 0.000 | 0.004 | 49.1 |
| Tenesa A (Israel) | 1.16 (1.12-1.20) | 0.000 | 0.003 | 50.3 |
| Tenesa A (Japan) | 1.16 (1.12-1.20) | 0.000 | 0.042 | 36.0 |
| Tenesa A (Kiel) | 1.16 (1.11-1.20) | 0.000 | 0.002 | 51.6 |
| Tenesa A (Spain) | 1.16 (1.11-1.20) | 0.000 | 0.002 | 50.9 |
| Pittman AM (CORGI) | 1.15 (1.11-1.19) | 0.000 | 0.003 | 50.6 |
| Pittman AM (DFCCS) | 1.15 (1.11-1.19) | 0.000 | 0.006 | 47.0 |
| Pittman AM (EPICOLON) | 1.15 (1.11-1.19) | 0.000 | 0.002 | 51.3 |
| Pittman AM (FCCPS) | 1.15 (1.11-1.19) | 0.000 | 0.002 | 50.9 |
| Pittman AM (MCCS) | 1.16 (1.11-1.20) | 0.000 | 0.002 | 51.1 |
| Pittman AM (NSCCG1) | 1.15 (1.11-1.19) | 0.000 | 0.003 | 50.0 |
| Pittman AM (NSCCG2) | 1.16 (1.11-1.20) | 0.000 | 0.003 | 50.5 |
| Pittman AM (VCQ) | 1.16 (1.11-1.20) | 0.000 | 0.002 | 51.5 |
| Middeldorp A | 1.15 (1.11-1.19) | 0.000 | 0.004 | 49.2 |
| Wijnen JT | 1.15 (1.11-1.19) | 0.000 | 0.002 | 51.6 |
| [Von Holst S](http://www.ncbi.nlm.nih.gov/pubmed?term=%22von%20Holst%20S%22%5BAuthor%5D) | 1.16 (1.12-1.20) | 0.000 | 0.002 | 50.7 |
| Xiong F | 1.14 (1.10-1.18) | 0.000 | 0.014 | 42.8 |
| [Talseth-Palmer BA](http://www.ncbi.nlm.nih.gov/pubmed?term=%22Talseth-Palmer%20BA%22%5BAuthor%5D) | 1.15 (1.11-1.20) | 0.000 | 0.002 | 51.5 |
| Ho JW | 1.16 (1.11-1.20) | 0.000 | 0.002 | 51.4 |
| Mates IN | 1.16 (1.11-1.20) | 0.000 | 0.002 | 50.8 |
| Current study | 1.14 (1.11-1.18) | 0.000 | 0.020 | 40.9 |

^a^DerSimonian and Laird Random-effects model used to determine the significance of the overall OR.

^b^Cochran’s *x*^2^-based *Q* statistic test used to assess the heterogeneity.
