# Supplementary material for: Replication Study in Chinese Population and Meta-Analysis Supports Association of the 11q23 Locus with Colorectal Cancer
Source: PLoS One. 2012 Sep 18;7(9):e45461. doi: 10.1371/journal.pone.0045461 (PMC3445543; doi:10.1371/journal.pone.0045461)
Supplement: Table S3 — Sensitivity analysis of the genotypic model of CC versus AA. (DOCX) [file pone.0045461.s005.docx]

**Table S3.** Sensitivity analysis of the genotypic model of CC vs. AA .

| Omitted study | OR(95%CI) | *P*(Z)^a^ | *P*^b^_heterogeneity_ | *I*^2^(%) |
| --- | --- | --- | --- | --- |
| Tenesa A (Scotland 1) | 1.29 (1.19-1.41) | 0.000 | 0.004 | 49.6 |
| Tenesa A (Scotland 2) | 1.32 (1.21-1.44) | 0.000 | 0.002 | 52.2 |
| Tenesa A (Canada) | 1.30 (1.19-1.42) | 0.000 | 0.002 | 52.4 |
| Tenesa A (DACHS) | 1.30 (1.19-1.43) | 0.000 | 0.001 | 53.2 |
| Tenesa A (England) | 1.32 (1.20-1.44) | 0.000 | 0.002 | 52.7 |
| Tenesa A (Israel) | 1.33 (1.22-1.44) | 0.000 | 0.003 | 50.2 |
| Tenesa A (Japan) | 1.34 (1.23-1.45) | 0.000 | 0.026 | 40.0 |
| Tenesa A (Kiel) | 1.32 (1.21-1.44) | 0.000 | 0.002 | 51.4 |
| Tenesa A (Spain) | 1.31 (1.20-1.43) | 0.000 | 0.001 | 53.4 |
| Pittman AM (CORGI) | 1.29 (1.19-1.41) | 0.000 | 0.003 | 50.1 |
| Pittman AM (DFCCS) | 1.30 (1.19-1.41) | 0.000 | 0.002 | 51.4 |
| Pittman AM (EPICOLON) | 1.30 (1.19-1.42) | 0.000 | 0.002 | 52.9 |
| Pittman AM (FCCPS) | 1.30 (1.20-1.42) | 0.000 | 0.001 | 53.3 |
| Pittman AM (MCCS) | 1.32 (1.21-1.44) | 0.000 | 0.003 | 51.5 |
| Pittman AM (NSCCG1) | 1.30 (1.19-1.43) | 0.000 | 0.002 | 52.8 |
| Pittman AM (NSCCG2) | 1.32 (1.21-1.44) | 0.000 | 0.002 | 51.9 |
| Pittman AM (VCQ) | 1.31 (1.20-1.43) | 0.000 | 0.001 | 53.4 |
| Wijnen JT | 1.31 (1.20-1.43) | 0.000 | 0.001 | 53.4 |
| [Von Holst S](http://www.ncbi.nlm.nih.gov/pubmed?term=%22von%20Holst%20S%22%5BAuthor%5D) | 1.31 (1.20-1.43) | 0.000 | 0.001 | 53.4 |
| Xiong F | 1.29 (1.18-1.40) | 0.000 | 0.008 | 46.8 |
| [Talseth-Palmer BA](http://www.ncbi.nlm.nih.gov/pubmed?term=%22Talseth-Palmer%20BA%22%5BAuthor%5D) | 1.31 (1.20-1.43) | 0.000 | 0.001 | 53.4 |
| Ho JW | 1.32 (1.21-1.44) | 0.000 | 0.002 | 52.9 |
| Mates IN | 1.32 (1.21-1.43) | 0.000 | 0.003 | 50.8 |
| Current study | 1.28 (1.19-1.39) | 0.000 | 0.014 | 43.5 |

^a^Z-test used to determine the significance of the overall OR.

^b^Cochran’s *x*^2^-based Q statistic test used to assess the heterogeneity.
