# Supplementary material for: Replication Study in Chinese Population and Meta-Analysis Supports Association of the 11q23 Locus with Colorectal Cancer
Source: PLoS One. 2012 Sep 18;7(9):e45461. doi: 10.1371/journal.pone.0045461 (PMC3445543; doi:10.1371/journal.pone.0045461)
Supplement: Table S4 — Sensitivity analysis of dominant model. (DOCX) [file pone.0045461.s006.docx]

**Table S4.** Sensitivity analysis of dominant model.

| Omitted study | OR(95%CI) | *P*(Z)^a^ | *P*^b^_heterogeneity_ | *I*^2^(%) |
| --- | --- | --- | --- | --- |
| Tenesa A (Scotland 1) | 1.20 (1.14-1.27) | 0.000 | 0.001 | 55.8 |
| Tenesa A (Scotland 2) | 1.20 (1.14-1.27) | 0.000 | 0.001 | 55.9 |
| Tenesa A (Canada) | 1.20 (1.14-1.26) | 0.000 | 0.001 | 55.1 |
| Tenesa A (DACHS) | 1.20 (1.14-1.27) | 0.000 | 0.001 | 55.5 |
| Tenesa A (England) | 1.21 (1.15-1.28) | 0.000 | 0.002 | 51.9 |
| Tenesa A (Israel) | 1.21 (1.15-1.27) | 0.000 | 0.001 | 55.5 |
| Tenesa A (Japan) | 1.22 (1.16-1.28) | 0.000 | 0.007 | 47.3 |
| Tenesa A (Kiel) | 1.20 (1.14-1.27) | 0.000 | 0.001 | 56.0 |
| Tenesa A (Spain) | 1.21 (1.15-1.27) | 0.000 | 0.001 | 54.7 |
| Pittman AM (CORGI) | 1.20 (1.14-1.27) | 0.000 | 0.001 | 56.0 |
| Pittman AM (DFCCS) | 1.19 (1.14-1.25) | 0.000 | 0.003 | 51.2 |
| Pittman AM (EPICOLON) | 1.20 (1.14-1.27) | 0.000 | 0.001 | 55.9 |
| Pittman AM (FCCPS) | 1.20 (1.14-1.26) | 0.000 | 0.001 | 55.4 |
| Pittman AM (MCCS) | 1.21 (1.14-1.27) | 0.000 | 0.001 | 56.0 |
| Pittman AM (NSCCG1) | 1.20 (1.14-1.27) | 0.000 | 0.001 | 55.1 |
| Pittman AM (NSCCG2) | 1.21 (1.15-1.27) | 0.000 | 0.001 | 55.2 |
| Pittman AM (VCQ) | 1.21 (1.15-1.27) | 0.000 | 0.001 | 54.8 |
| Wijnen JT | 1.20 (1.14-1.27) | 0.000 | 0.001 | 55.9 |
| [Von Holst S](http://www.ncbi.nlm.nih.gov/pubmed?term=%22von%20Holst%20S%22%5BAuthor%5D) | 1.21 (1.15-1.28) | 0.000 | 0.001 | 53.5 |
| Xiong F | 1.19 (1.13-1.25) | 0.000 | 0.009 | 46.0 |
| [Talseth-Palmer BA](http://www.ncbi.nlm.nih.gov/pubmed?term=%22Talseth-Palmer%20BA%22%5BAuthor%5D) | 1.21 (1.15-1.27) | 0.000 | 0.001 | 55.7 |
| Ho JW | 1.20 (1.14-1.27) | 0.000 | 0.001 | 55.8 |
| Mates IN | 1.21 (1.15-1.27) | 0.000 | 0.001 | 55.9 |
| Current study | 1.18 (1.14-1.24) | 0.000 | 0.040 | 37.0% |

^a^Z-test used to determine the significance of the overall OR.

^b^Cochran’s *x*^2^-based Q statistic test used to assess the heterogeneity.
