# Supplementary material for: Replication Study in Chinese Population and Meta-Analysis Supports Association of the 11q23 Locus with Colorectal Cancer
Source: PLoS One. 2012 Sep 18;7(9):e45461. doi: 10.1371/journal.pone.0045461 (PMC3445543; doi:10.1371/journal.pone.0045461)
Supplement: Table S5 — Sensitivity analysis of recessive model. (DOCX) [file pone.0045461.s007.docx]

**Table S5.** Sensitivity analysis of recessive model.

| Omitted study | OR(95%CI) | *P*(Z)^a^ | *P*^b^_heterogeneity_ | *I*^2^(%) |
| --- | --- | --- | --- | --- |
| Tenesa A (Scotland 1) | 1.18 (1.10-1.27) | 0.000 | 0.082 | 30.7 |
| Tenesa A (Scotland 2) | 1.21 (1.13-1.30) | 0.000 | 0.043 | 36.4 |
| Tenesa A (Canada) | 1.20 (1.11-1.28) | 0.000 | 0.032 | 38.4 |
| Tenesa A (DACHS) | 1.20 (1.11-1.29) | 0.000 | 0.027 | 39.6 |
| Tenesa A (England) | 1.20 (1.11-1.29) | 0.000 | 0.026 | 39.8 |
| Tenesa A (Israel) | 1.21 (1.13-1.30) | 0.000 | 0.049 | 35.3 |
| Tenesa A (Japan) | 1.22 (1.14-1.31) | 0.000 | 0.119 | 26.6 |
| Tenesa A (Kiel) | 1.21 (1.13-1.30) | 0.000 | 0.045 | 36.1 |
| Tenesa A (Spain) | 1.20 (1.12-1.29) | 0.000 | 0.026 | 39.9 |
| Pittman AM (CORGI) | 1.18 (1.11-1.27) | 0.000 | 0.078 | 31.2 |
| Pittman AM (DFCCS) | 1.19 (1.11-1.28) | 0.000 | 0.034 | 38.1 |
| Pittman AM (EPICOLON) | 1.19 (1.11-1.28) | 0.000 | 0.030 | 38.9 |
| Pittman AM (FCCPS) | 1.20 (1.11-1.29) | 0.000 | 0.027 | 39.7 |
| Pittman AM (MCCS) | 1.21 (1.13-1.29) | 0.000 | 0.047 | 35.7 |
| Pittman AM (NSCCG1) | 1.19 (1.11-1.29) | 0.000 | 0.030 | 39.0 |
| Pittman AM (NSCCG2) | 1.21 (1.12-1.30) | 0.000 | 0.033 | 38.3 |
| Pittman AM (VCQ) | 1.19 (1.11-1.28) | 0.000 | 0.030 | 38.9 |
| Wijnen JT | 1.20 (1.12-1.29) | 0.000 | 0.027 | 39.8 |
| [Von Holst S](http://www.ncbi.nlm.nih.gov/pubmed?term=%22von%20Holst%20S%22%5BAuthor%5D) | 1.20 (1.11-1.29) | 0.000 | 0.027 | 39.7 |
| Xiong F | 1.19 (1.10-1.28) | 0.000 | 0.041 | 36.7 |
| [Talseth-Palmer BA](http://www.ncbi.nlm.nih.gov/pubmed?term=%22Talseth-Palmer%20BA%22%5BAuthor%5D) | 1.20 (1.12-1.29) | 0.000 | 0.026 | 39.9 |
| Ho JW | 1.21 (1.13-1.30) | 0.000 | 0.045 | 36.0 |
| Mates IN | 1.20 (1.12-1.29) | 0.000 | 0.048 | 35.4 |
| Current study | 1.19 (1.11-1.28) | 0.000 | 0.038 | 37.2 |

^a^Z-test used to determine the significance of the overall OR.

^b^Cochran’s *x*^2^-based Q statistic test used to assess the heterogeneity.
